# Progress in General Surgery

**Published:** 1902-05-17

**Authors:** 


### Progress in General Surgery.

(*Continued from page* 49.)

**Fractures and Dislocations.—Upper Extremity.**—A method of reduction of shoulder dislocations which is practically new, although it is to a certain extent adapted and modified from older procedures, has recently[8] been advocated by F. Hofmeister, of Tubingen. The plan is simple, and requires only a vertical extension apparatus and some weights up to about 20 kilograms. The patient is laid upon the uninjured side, and the injured arm is bandaged as high as the insertion of the deltoid in order to secure a firm hold for the extension cord. The latter is passed over the pulley, and a weight of about five kilograms (11 lb.) is attached. This holds the limb in complete vertical extension, and exerts a continuous pull against the muscles of the shoulder girdle. After a few minutes more weight is added, and this process is kept up until the contraction of the muscles is overcome, and the humeral head is drawn to the edge of the glenoid cavity, into which it either slips spontaneously or may be pushed very easily. The time required is from 15 to 45 minutes, and the weight from 5 to 20 kilograms. The advantages claimed are avoidance of anæsthesia, simplicity, and entire absence of danger; moreover, Kocher's method is not always successful. For all ordinary forms of shoulder dislocation this new method is well adapted and worthy of trial. Hofmeister has employed it successfully in seven cases, although in three of these the injury was more than eight days old. In his excellent paper upon the recent advances in the diagnosis and treatment of fractures of the upper extremity J. E. Platt[9] truly says that oblique intra-articular fractures of the condyles are the most frequent fractures of the lower end of the humerus. Out of 60 consecutive cases which came under his personal notice 27 were of this nature. Most of the patients were children. The external condyle is much more frequently involved than the internal. An examination of specimens has shown that the injury in the case of the external condyle may be a true fracture running from the neighbourhood of the external epicondyle to the articular surface, or a separation of the epiphysial centres of the capitellum and external epicondyle, or partly a fracture and partly an epiphysial separation. In the case of the internal condyle in adults the injury would appear to be always a true fracture running from the neighbourhood of the internal epicondyle to the articular surface. As regards operative treatment of fractures of the lower end of the humerus, Platt recommends it in certain cases, more particularly in T-shaped and comminuted fractures, which are liable to leave a great deal of stiffness and permanent deformity. He advises the same treatment in cases where it is found to be impossible to reduce the displacement of the fragments by external manipulation. Dr. Chas. McBurney describes[10] an operation he performed for old posterior dislocation of the forearm. The patient, aged 17, fell downstairs nine weeks previously and injured her left elbow. The injury had been regarded as a fracture and treated as such, the arm being placed in the extended position with long anterior splints. It was evident that a posterior dislocation existed, and the X-ray photograph confirmed this, and proved the non-existence of fracture at any point. Very slight motion could be made at the elbow. Under ether vigorous attempts were made to reduce the dislocation but completely failed. An incision five inches long, parallel with the long axis of the arm, was made, the centre of the incision crossing the internal condyle. All the soft parts were divided down to the bones and the ulnar nerve and tissues pushed backwards. The internal lateral ligament was separated from the condyle, the joint opened, and the sigmoid cavity of the olecranon freed from new tissue. It was still found impossible to produce reduction. A short incision was therefore made on the outer side of the elbow, and the soft parts and lateral ligament freely divided. Complete reduction was now made, the wounds sutured and arm flexed to less than a right angle in plaster of Paris dressing. The dressings were changed on seventh day, when the wounds were completely healed. Passive movement was now commenced. The ultimate result was that voluntary flexion, pronation, and supination were entirely complete and normal. Dr. J. B. Roberts[11] puts on record a case of gangrene of the forearm after an open fracture of the radius and ulna, which appears to have been due to infection by the bacillus aërogenes capsulatus. A young girl, aged 12, slipped and fell, sustaining a fracture of the left radius and ulna about the junction of the middle and lower thirds. Although the wound, which was a small one, was cleansed with soap and water and perchloride solution (1 in 1,000), the hand in three days became rapidly bluish black and cold, and the discoloration extended up nearly to the elbow. The forearm was swollen, tense, and crepitated on pressure. The temperature was 103·8° Fahr., and the urine contained albumen with casts. Amputation was performed above the elbow, above all gangrenous structures. A little gangrene occurred in one flap, but the child was discharged from the hospital on the twentieth day with the wound almost healed. On dissection there was found not much fluid in the tissues and not a great deal of gas; the most intense gangrene was near the small wound. The arteries had clot in them and also contained gas. The gas in the radial and ulnar arteries formed long bubbles, with smaller areas of blood clots in between, giving a beaded appearance. The difficulty in the proper reduction of the displaced fragments in Colles's fracture is usually attributed to impaction, which causes fixation of the fragments one to the other. However, Joseph Griffiths[12] shows that in the majority of instances impaction, such as is enough to defy manipulation with or without an anæsthetic, does not occur, and that it is not difficult to set the fracture properly. He finds that a simple and full flexion forwards at the wrist-joint without any pulling suffices to reduce the deformity and to give back the natural contour to the broken radius; by letting the hand go the fragments at once regain their displaced position. From an examination of specimens he has come to the conclusion that there is no important displacement in an outward and upward direction of the lower fragment. He is convinced, as was Dr. Alex. Gordon, that the small amount of impaction can with ease be overcome by manipulation with or without an anæsthetic. The "give" which accompanies traction or pulling on the hand is due to the laxity of the anterior and posterior ligaments of the first and second rows of carpal bones, and to the extension of the muscles (the flexors and extensors) of the forearm and wrist. But when the hand is bent forwards, the extensors readily yield and the dorsal ligaments of the wrist and mid-carpal joints are soon put on the stretch, and further pull upon them by further flexion is communicated to the turned-back fragment of the radius, to which the posterior ligament is attached, causing it to yield and turn forwards to its proper position, all deformity thereby disappearing. The hand can be easily fixed in this flexed position upon an ordinary Carr's splint by placing a large pad in front of the lower end of the shaft of the radius and strapping the forearm and hand to the splint, a dorsal splint being unnecessary. Dr. Griffiths has designed a light, comfortable, and not expensive splint. With a little padding it can be made to fit the forearm of almost all adults. It may be constructed of copper, brass, or aluminium, and fits pretty accurately on the front of the lower part of forearm and palm when the wrist is fully flexed, with a strap across back of upper part of forearm and a Y-shaped strap across the metacarpals, one piece of which going above, the other below the thumb. After fourteen days the splint is taken off and the wrist freely moved; this is again done seven days later. At the end of four weeks the splint is discarded and free use of the hand allowed. At the recent meeting of the Royal Academy of Medicine in Ireland Dr. Knott[13] exhibited an interesting specimen of Colles's fracture in which the displacement was to the opposite side to that which usually takes place. These cases have been recently investigated by Dr. J. B. Roberts, of Philadelphia, in his monography on fractures of the lower end of the radius. An instance of simple forward dislocation of the semilunar bone is recorded by Dr. C. H. Catherwood[14] in a man aged 48 who fell downstairs on to his hand, which was in the over-extended position. A diagnosis of Colles's fracture was at first made, but next day the following signs were found: considerable flexion and fixation of fingers, tenderness over radial side of wrist-joint, almost complete loss of function of the hand, and on the anterior surface of the radio-carpal articulation a large but well-fixed piece of bone, together with a distinct depression on the dorsum of the wrist where the semilunar bone should be. The X-ray picture displayed the dislocation well, and a large piece broken off the lower end of the radius. Operative interference and removal of the displaced semilunar bone was suggested but refused by the patient.

[8] New York Med. Rec., Aug. 3, 1901, p. 177, and Beiträge zur klin. Chirurgie, Bd. xxx., Sept. 2, 1901. [9] Practitioner, Aug., 1901., p. 205. [10] New York Med. Rec., Jan. 18, 1902, p. 114. [11] Annals of Surgery, June, 1901, p. 768. [12] Brit. Med. Jour., Dec. 7, 1901, p. 1654. [13] Brit. Med. Jour., April 19, 1902, p. 965. [14] New York Med. Rec., Sept. 7, 1901, p. 392.

